# Preclinical Simulation of Transoral Robotic Thyroidectomy Using a Training Box and the Versius Robotic Platform

**DOI:** 10.1002/oto2.70309

**Published:** 2026-09-28

**Authors:** Guilherme R. Agne, Valdenor N. Feitosa, Antonio Bertelli, Rafael de Cicco, Luiza H. C. da Silva, Leandro L. Matos, Bruno M. Murara, Renan B. Lira, Everson L. de Almeida Artifon, Luiz P. Kowalski

**Affiliations:** ^1^ Oncology Postgraduate Program, Faculdade de Medicina FMUSP Universidade de São Paulo São Paulo São Paulo Brazil; ^2^ Department of Head and Neck Surgery PESCOP Group Balneário Camboriú Santa Catarina Brazil; ^3^ Department of Head and Neck Surgery Irmandade Santa Casa de Misericórdia de Fortaleza Fortaleza Ceará Brazil; ^4^ Head and Neck Surgery Division, Department of Surgery Faculty of Medical Sciences of Santa Casa de São Paulo São Paulo São Paulo Brazil; ^5^ Department of Surgery Instituto de Cancer Doctor Arnaldo São Paulo São Paulo Brazil; ^6^ Department of Head and Neck Surgery, School of Medicine Universidade de São Paulo São Paulo São Paulo Brazil; ^7^ Instituto de Ensino e Pesquisa Albert Einstein Faculdade Israelita de Ciencias da Saude Albert Einstein São Paulo São Paulo Brazil; ^8^ Department of Otorhinolaryngology PESCOP Group Balneário Camboriú Santa Catarina Brazil; ^9^ Department of Head and Neck Surgery Hospital Israelita Albert Einstein São Paulo São Paulo Brazil; ^10^ Department of Head and Neck Surgery and Otorhinolaryngology AC Camargo Cancer Center São Paulo São Paulo Brazil; ^11^ Department of Surgery Universidade de São Paulo São Paulo São Paulo Brazil

**Keywords:** minimally invasive training, robotic thyroidectomy, surgical simulation, transoral surgery, Versius robotic system

## Abstract

Transoral robotic thyroidectomy (TORT) represents an alternative to open surgery, offering favorable cosmetic outcomes and comparable oncologic results. However, standardized training pathways and validated simulation models replicating standardized setups for emerging robotic platforms remain limited. An experimental simulation of total thyroidectomy and central (recurrent) neck dissection was performed with the Versius robotic system in a validated synthetic training box. Optimal robotic positioning and docking were determined, and a standardized procedural sequence was followed to ensure optimal results. Simulation was successful only with a C‐shaped configuration of the robotic arms and a posteriorly positioned camera in a Z‐shaped layout, matching prior cadaveric findings. All thyroidectomy steps proceeded smoothly. However, during central neck dissection, instrument collisions limited access to deeper spaces, restricting maneuverability. The training box allowed successful simulation of TORT with the Versius system in this proof‐of‐concept study, providing a platform for evaluating robotic positioning and docking configurations and supporting further investigation of its potential applications in training and preclinical assessment.

Transoral Endoscopic Thyroidectomy Vestibular Approach (TOETVA) has gained increasing acceptance in Western countries due to its ability to avoid visible cutaneous scarring, its reproducibility, and its relatively short learning curve.^1^ This procedure may also be performed with robotic assistance, known as Transoral Robotic Thyroidectomy (TORT). Previous studies have demonstrated that complication rates and oncologic outcomes are comparable to those of conventional open thyroidectomy.^2^ Nevertheless, as with open surgery, outcomes are strongly influenced by surgeon training and case volume. The learning curve for TOETVA has been estimated to range from 10 to 20 cases.^3^

Despite the growing adoption of remote‐access thyroidectomy techniques, no consensus currently exists regarding standardized training pathways for surgeons performing either endoscopic or robotic approaches. Furthermore, the published literature addressing training strategies for the introduction and adaptation of new robotic platforms in thyroid surgery remains scarce. Several studies have employed cadaveric models for surgeon training in these procedures. In 2024, a training box designed to simulate the key steps of TOETVA and central (recurrent) neck dissection was developed and validated using synthetic materials.^4^ Compared with cadaveric training, this simulation model offers important practical advantages, including lower cost, greater accessibility, and the possibility of repeated use, thereby allowing standardized practice without the logistical, ethical, and financial constraints associated with human specimens.

The Versius robotic system was introduced in 2019 and is currently undergoing development for applications in head and neck surgery. A preclinical feasibility study using cadaveric models evaluated the transvestibular approach with this robotic platform and established an optimal standardized configuration.^5^ The present study aims to evaluate a training box for TORT simulation using the Versius robotic system.

## Methods

An experimental study simulated total thyroidectomy and central neck dissection using the Versius robotic system in a validated training box. Seven head and neck surgeons experienced and certified in robotic surgery participated in the experimental evaluation. A series of preliminary tests determined optimal positioning and docking configuration. The system was equipped with a 30° endoscopic camera, scissors in the right arm, and a Maryland dissector in the left arm.

A standardized sequence defined by the training protocol was followed. The procedure began with elevation of subplatysmal flaps, followed by midline dissection and isthmotomy. The superior thyroid lobe was dissected from the superior parathyroid gland. The recurrent laryngeal nerve was identified and dissected, allowing completion of lobectomy. After repeating on the contralateral side, the nerve was followed caudally and central neck dissection performed.

This study was approved by the Institutional Review Board (IRB) of the University of São Paulo (protocol number 7.421.889).

## Results

### Robotic Configuration and Simulation Assessment

The optimal robotic configuration was determined after multiple preliminary simulation attempts evaluating different docking arrangements.

The lateral robotic arms were docked with their central axes aligned to the mentum of the simulator and positioned 40 cm laterally from the midline on each side. Both arms were configured in a C‐shaped arrangement, with elbow and wrist articulations adjusted to direct the trocar trajectories toward the hyoid bone.

The camera arm was positioned cranially, 40 cm above the mentum, with slight lateral displacement. Its elbow and wrist articulations were configured in a Z‐shaped arrangement, allowing the camera to be directed toward the hyoid region.

The position of the camera arm and its docking angle did not significantly influence instrument collisions or procedural performance. In contrast, the configuration of the lateral working arms proved critical. Proper angulation of these arms was necessary to maximize the distance between the robotic wrists, thereby minimizing external collisions and optimizing instrument maneuverability throughout the procedure (Figure 1).

**Figure 1:**
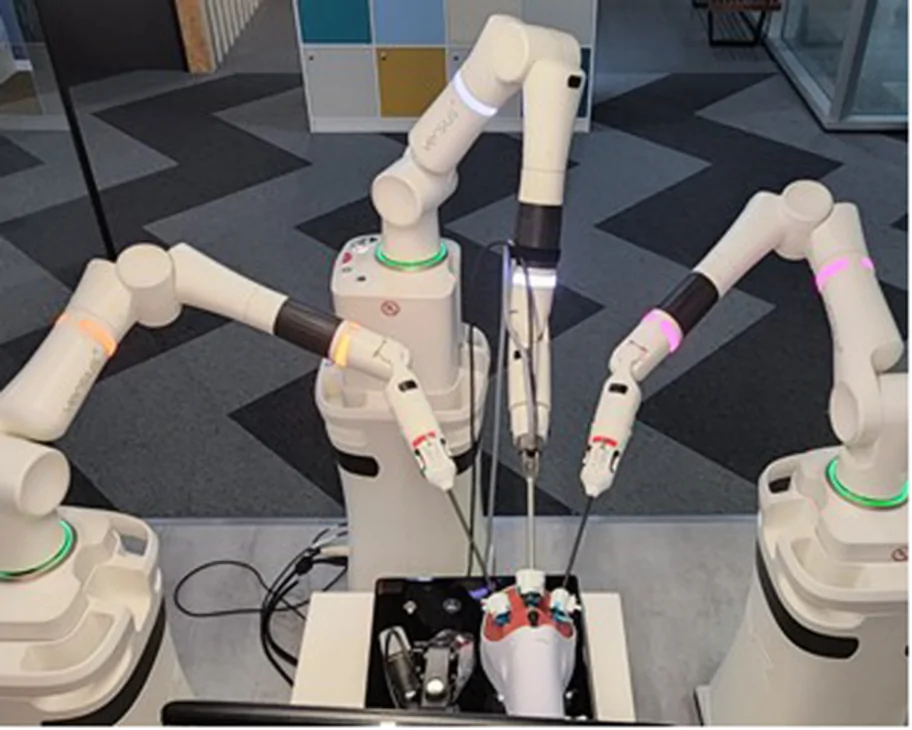
Optimal robotic configuration for transoral thyroidectomy simulation. The Versius system demonstrates a C‐shaped arrangement of the robotic arms with posterior camera positioning in a Z‐shaped layout, replicating the standardized setup validated in cadaveric studies.

Successful completion was defined as the ability to complete all planned procedural steps, including subplatysmal flap elevation, midline dissection, isthmotomy, bilateral lobectomy, identification of the recurrent laryngeal nerves, and central neck dissection, without requiring repositioning of the robotic system or conversion to an alternative configuration.

The training box enabled simulation of the thyroidectomy steps using synthetic tissues that accurately mimicked the anatomical structures (Figure 2).

**Figure 2:**
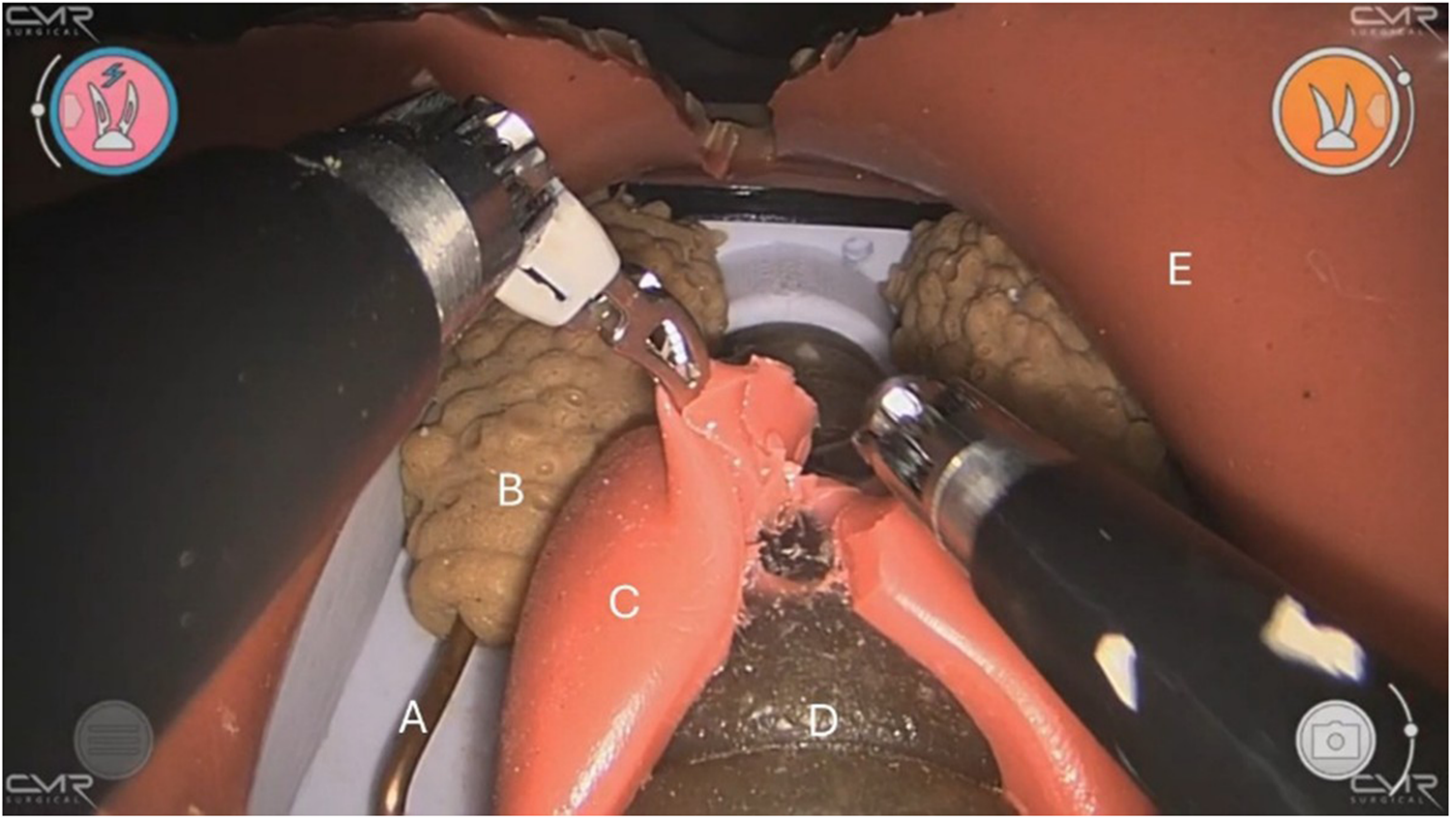
Intraoperative robotic visualization of the synthetic anatomical structures within the training box. (A) Recurrent laryngeal nerve, (B) level VI lymph nodes, (C) thyroid gland, (D) trachea, (E) strap muscles.

During the simulation of central neck dissection, which involves a deeper and more confined working space within the training box, progressive and restrictive collisions between the robotic arms were observed (Figure 3).

**Figure 3:**
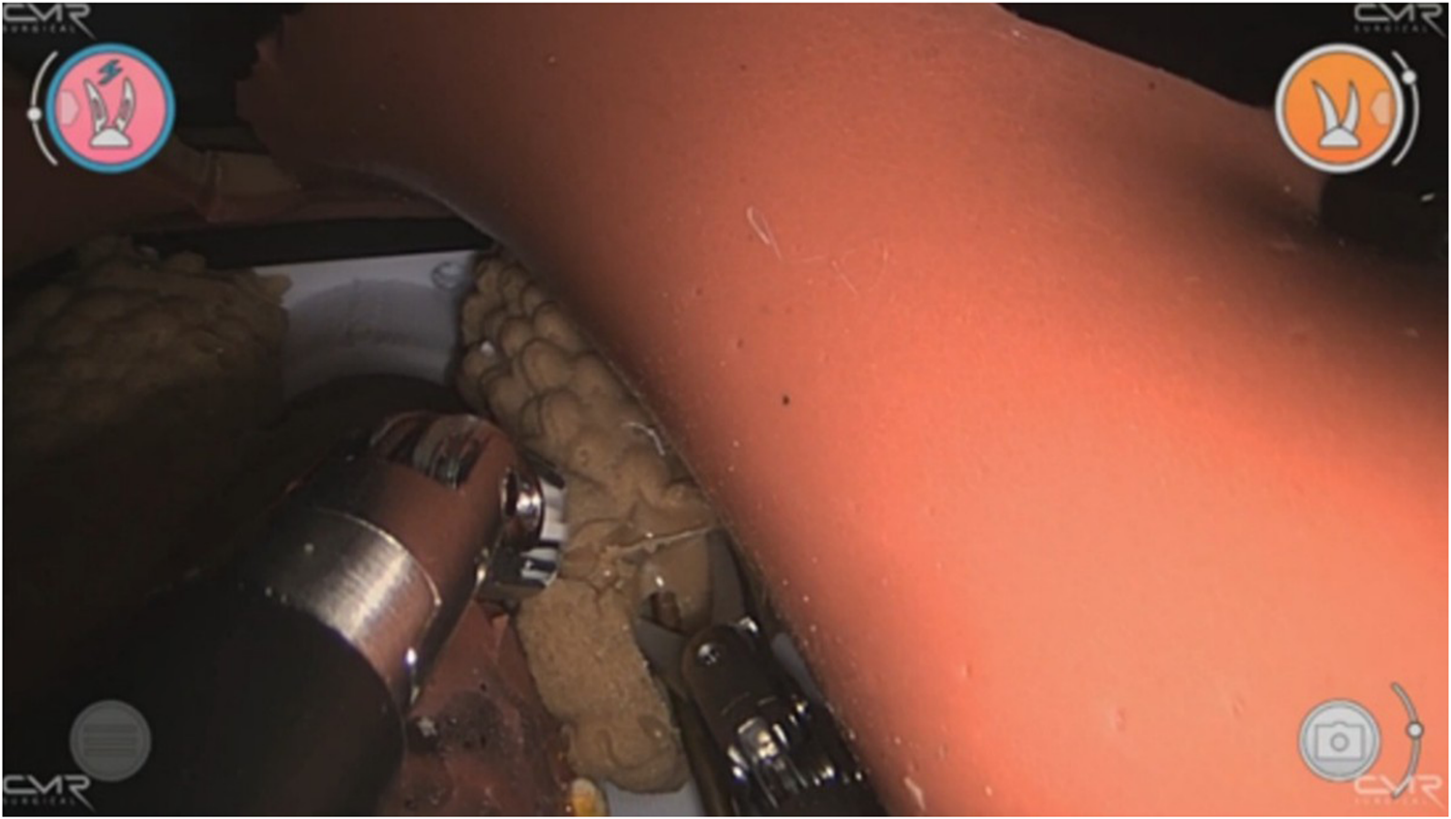
Console view during paratracheal (level VI) neck dissection, demonstrating the deeper operative field and confined working space.

### Assessment of Instrument Collisions

Instrument collisions were observed during simulation of the central (recurrent) neck dissection. This stage of the procedure was performed at a depth ranging from 16 to 20 cm from the vestibular incision plane. Collisions became evident during this phase and ultimately prevented further progression of the dissection within the distal third of the central neck compartment (Figure 4). At this stage, the estimated triangulation angle between the lateral trocar, camera, and the distal portion of the dissection field was approximately 81.6°.

**Figure 4:**
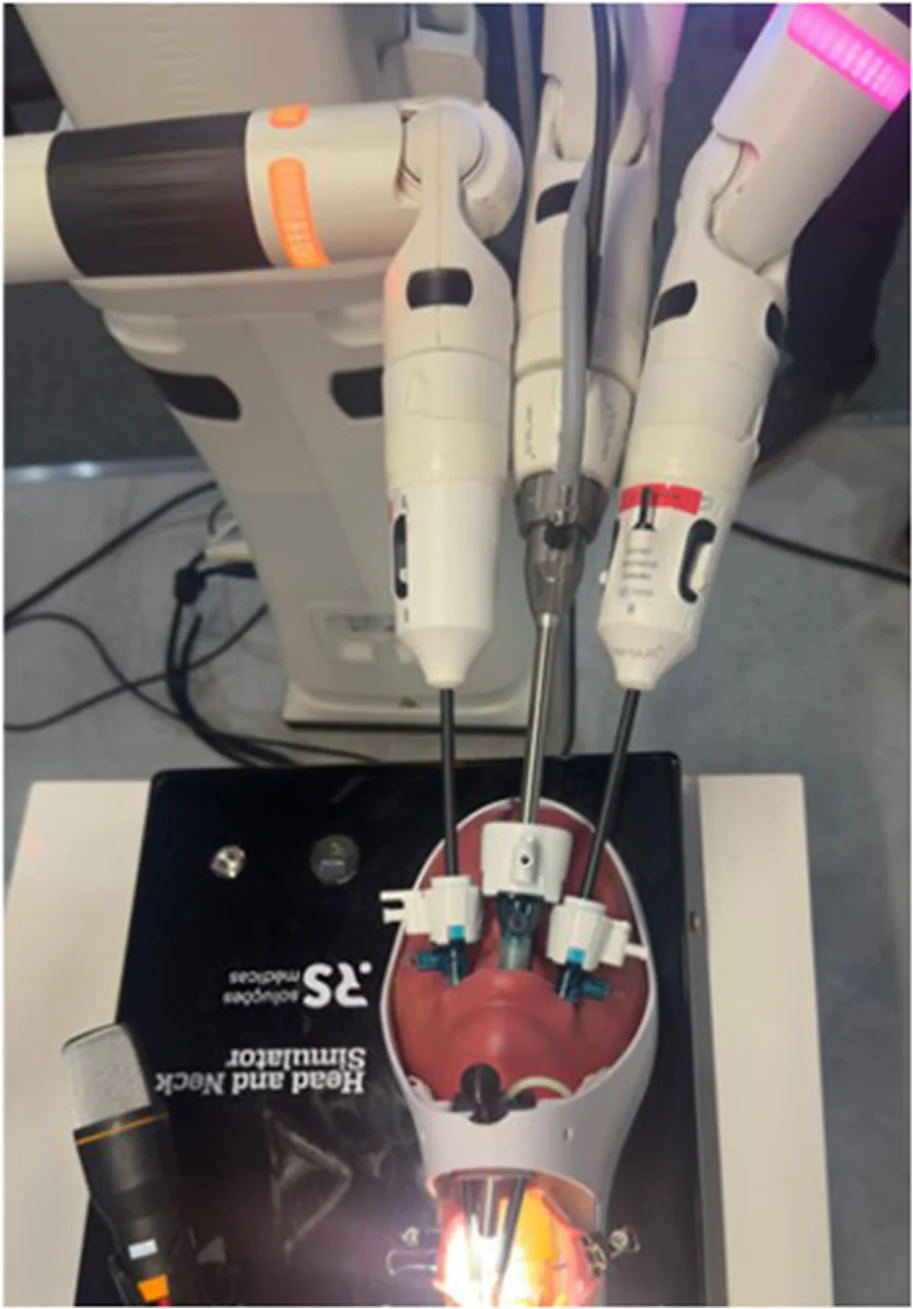
Progressive convergence of the robotic arms during distal dissection, illustrating the “funnel effect” with resultant instrument collisions and compromised maneuverability.

In addition to operative depth, the fixed distance between the vestibular ports may have contributed to this phenomenon. In the training model, the lateral trocars were positioned 2.75 cm from the central trocar on each side. As dissection advanced into deeper operative planes, the combination of fixed entry points and increasing depth progressively reduced the working angle between the instruments, resulting in convergence of the robotic arms and decreased maneuverability.

## Discussion

The training box successfully simulated TORT with the Versius system in this proof‐of‐concept exercise, demonstrating that the model can be used to explore robotic positioning and docking strategies in a controlled environment. This is particularly important given that one of the main challenges to the widespread adoption of this technique is the limited availability of structured training and simulation models.

The novelty of the present study lies not in demonstrating the feasibility of the Versius platform itself, which has already been reported in our previous cadaveric investigation, but in evaluating whether the same robotic configuration can be reproduced in a validated synthetic simulation model. Compared with cadaveric specimens, the training box offers potential advantages including lower cost, wider accessibility, and the possibility of repeated procedural practice without the logistical constraints associated with anatomical laboratories.

Furthermore, as multiple new robotic platforms are currently under development, the anatomical fidelity of the training box and its ability to reproduce key procedural steps may make it a useful platform for exploratory assessment of robotic configurations in emerging systems. Although cadaveric studies remain the primary method for evaluating new platforms, the training box may represent an accessible adjunct for preliminary assessment of robotic positioning and docking strategies, warranting further validation studies. Moreover, the ability to accurately reproduce the procedural steps performed in cadaveric models reinforces the anatomical fidelity and potential educational value of the training box as a simulation platform for robotic systems. Traditionally, head and neck surgeons possess extensive expertise in open procedures, yet often have limited exposure to laparoscopic instruments and robotic approaches. Consequently, there is a demand for high‐fidelity training modalities that offer greater accessibility than cadaveric specimens. To date, virtual reality platforms specifically designed for simulating remote‐access thyroidectomy remain unavailable.

An additional finding was the occurrence of progressive instrument collisions during deep central neck dissection. As dissection progresses into deeper operative planes while maintaining fixed entry points, the working angles between instruments progressively decrease, resulting in convergence of the robotic arms and reduced maneuverability, a phenomenon commonly described as the “funnel effect.”

The funnel effect observed during deep central neck dissection appears to be primarily related to geometric constraints rather than exclusively to the robotic platform or the simulation model. With fixed vestibular entry points separated by 2.75 cm from the central port, progressive advancement into deeper operative planes inherently reduces the angle formed between the working instruments.

This phenomenon was not observed in our previous cadaveric study using the same robotic configuration. However, direct comparison is limited because an equivalent recurrent (central) neck dissection was not performed in the cadaveric model. Consequently, it remains unclear whether the observed funnel effect reflects a limitation of the Versius platform when operating in deeper confined spaces, geometric constraints imposed by the synthetic simulator, or a combination of both factors. One potential strategy to mitigate this phenomenon is to increase the lateral spacing between the trocar entry points, thereby improving instrument triangulation and reducing arm convergence during deep dissection.

Future investigations incorporating comparable central neck dissections in cadaveric and clinical settings will be necessary to better define the origin and significance of this finding. Interestingly, the identification of this phenomenon may represent one of the advantages of simulation‐based testing, as it allows potential geometric constraints to be recognized before clinical implementation.

Careful patient selection during early clinical phases is essential when transitioning from preclinical to clinical application. This approach allows for the progressive identification of the true operational limits of the robotic system and the development of strategies aimed at optimizing its performance in anatomically constrained scenarios.

## Conclusion

This proof‐of‐concept simulation demonstrated that the training box can be used to perform the main steps of TORT with the Versius robotic system and to evaluate robotic positioning and docking configurations. These preliminary findings support further studies to determine the role of this model in robotic training and preclinical assessment of emerging platforms.

## Author Contributions

**Guilherme R. Agne**, conceptualization, data curation, formal analysis, investigation, methodology, project administration, resources, validation, writing original draft; **Valdenor N. Feitosa**, resources, validation; **Antonio Bertelli**, methodology, writing original draft; **Rafael de Cicco**, methodology, writing original draft; **Luiza H. C. da Silva**, resources, validation; **Leandro L. Matos**, methodology, writing original draft; **Bruno M. Murara**, methodology, writing original draft; **Renan B. Lira**, conceptualization, formal analysis; **Everson L. de Almeida Artifon**, resources, validation; **Luiz P. Kowalski**, conceptualization, formal analysis, validation.

## Disclosures

### Competing interests

Guilherme Reimann Agne, Valdenor Neves Feitosa, Rafael de Cicco, Luiza Helena Crispim da Silva, Bruno Matos Murara, Renan Bezerra Lira, Everson Luiz de Almeida Artifon and Luiz Paulo Kowalski declare they have no financial interests. Leandro Luongo Matos has received speaker fees from Company MSD. Antonio Bertelli has received speaker fees and recipient of travel grants from Company Johnson & Johnson and Company Merck. Proctor in robotic surgery.

### Funding source

The authors declare that no funds, grants, or other support was received during the preparation of this manuscript.
